# Supplementary material for: Constraining composition and temperature variations in the mantle transition zone
Source: Nat Commun. 2022 Mar 1;13:1094. doi: 10.1038/s41467-022-28709-7 (PMC8888558; doi:10.1038/s41467-022-28709-7)
Supplement: Supplementary file 1 — Supplementary Information [file 41467_2022_28709_MOESM1_ESM.pdf]

Supplementary Information

Constraining Composition and Temperature Variations in the Mantle Transition Zone

Wen-Yi Zhou[1,2*], Ming Hao[1,2], Jin S. Zhang[1,2*], Bin Chen[3], Ruijia Wang[1],

Brandon Schmandt[1]

1. Department of Earth and Planetary Sciences, University of New Mexico, Albuquerque, NM, USA

2. Institute of Meteoritics, University of New Mexico, Albuquerque, NM, USA

3. Hawaii Institute of Geophysics and Planetology, University of Hawaiʻi at Mānoa, Honolulu, HI, USA

*Corresponding author: Wen-Yi Zhou (zwy1993@unm.edu); Jin Zhang (jinzhang@unm.edu)

Supplementary Discussion 1: Testing the robustness of the wadsleyite proportion, temperature anomaly, and water content models given uncertainties of the mineral physics models used in this study

In terms of the Fe and water effects on the *V*p and *V*s of wadsleyite, the average difference between model-predicted values and the experimental data from previous studies is 0.7%[1]. In terms of the *T* effect, as shown in Fig. 1 in the main text, the average difference between the model-predicted values and the experimental data is even less and on the level of 0.1%. However, given the limited *T* range we experimentally explored, in the parameter space used in this study (ΔT from -150K to 150 K compared with 1870 K[2], water content from 0 to 1 wt%), the calculated *V*p and *V*s uncertainties of our wadsleyite sample at 1870 K and 14 GPa are higher than 0.1%, and on the level of 1% without considering the composition effect-induced uncertainties. Therefore, as a conservative estimate, we assigned an assumed 1 standard deviation of 10% for each of the parameters (*a*, *b*, *c*, *d*, *A*, *B*, *C*, *D*, *e*, and *f* in Equation 1-5 in Methods) used in our modeling work, and then took the following procedures to evaluate the possible influence of these uncertainties on the robustness of our calculation:

1) Instead of fixing the *a*, *b*, *c*, *d*, *A*, *B*, *C*, *D*, *e*, and *f* parameters to the best-fit values, we randomly sampled the value of each parameter from a normal (Gaussian) distribution based on the best-fit value and the assumed 10% standard deviation.

2) We then re-calculated the global wadsleyite proportion, ΔT, and water content maps in the upper MTZ.

3) We repeated setup 1) and 2) a few times, and found that they yielded nearly identical results compared to those shown in the manuscript, which suggests that these uncertainties are unlikely to affect the robustness of this study. The simple explanation is that the influence of the global

variance in seismic structure is large compared to the uncertainties in the experimentally derived model parameters. Two typical test results are shown in Supplementary Fig. 9 and Supplementary Table 7.

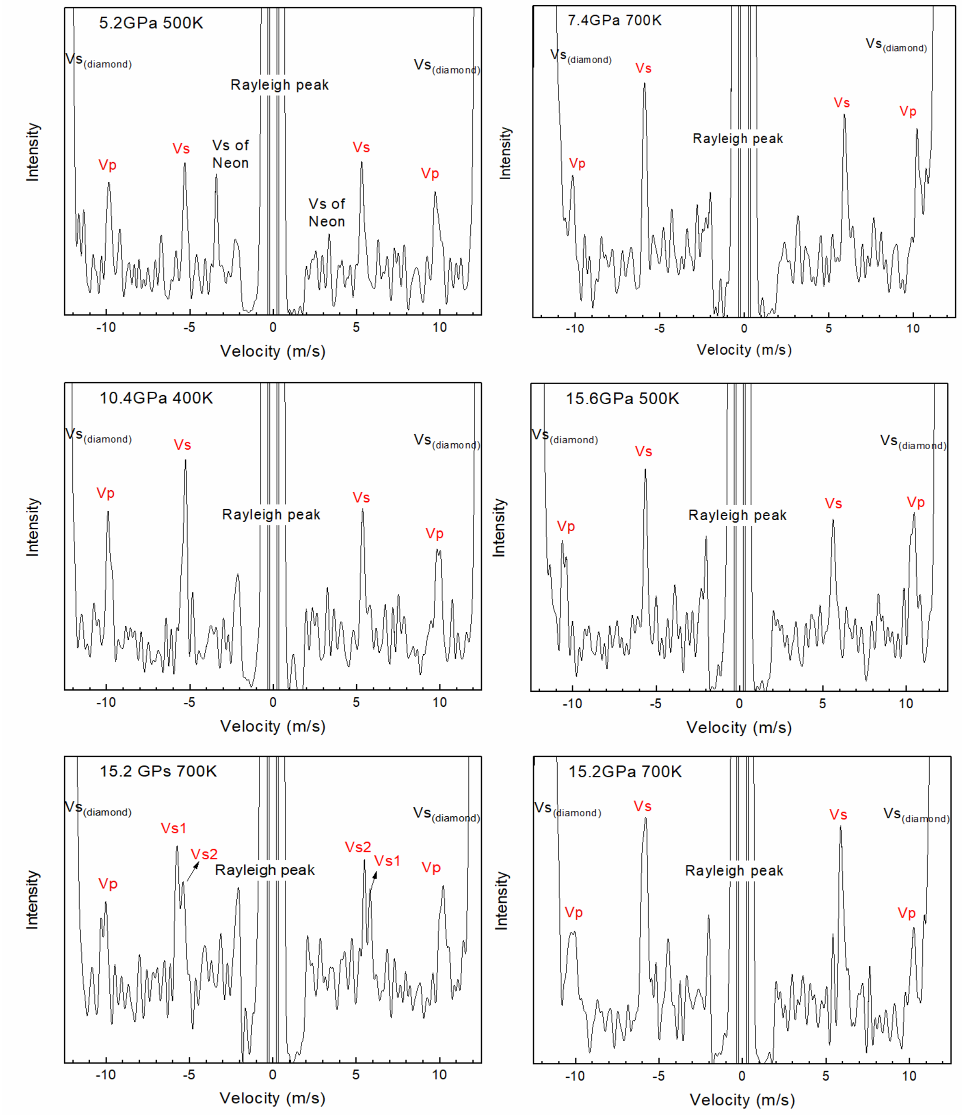

Supplementary Figure 1. Representative Brillouin spectra of single-crystal wadsleyite at different high *P*-*T* conditions. The spectra have been processed with a low pass filter. The collection time is approximately 20 minutes for each spectrum.

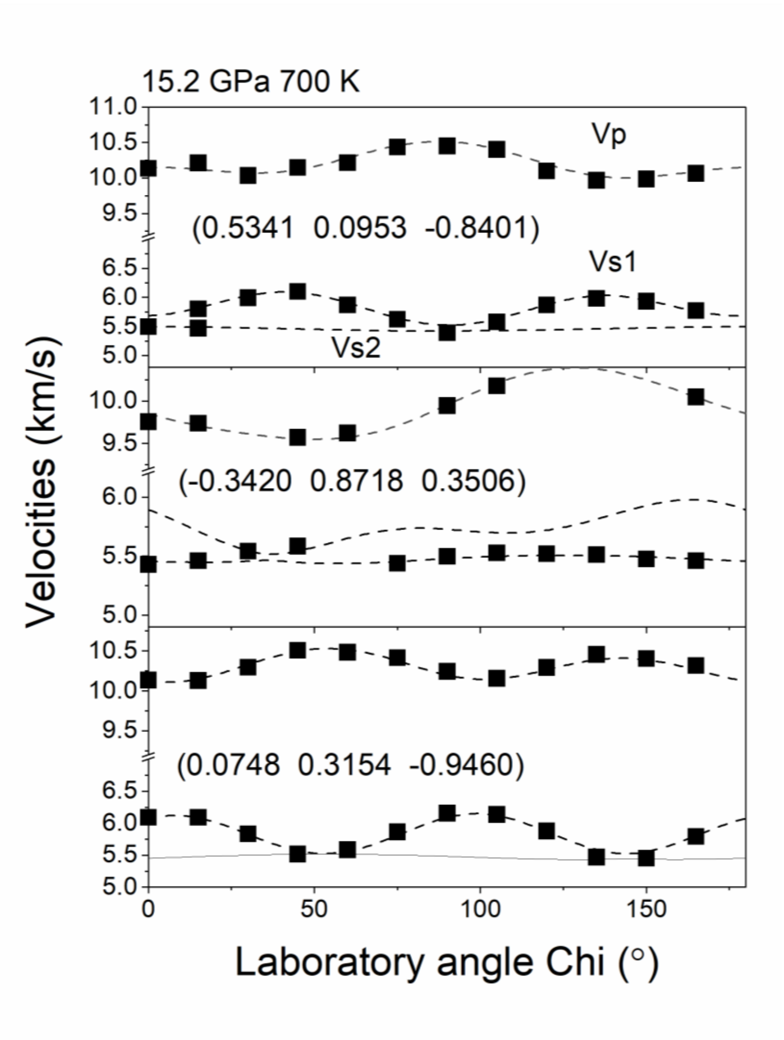

Supplementary Figure 2. Change of velocities as a function of laboratory measurement angle Chi at 15.2 GPa and 700 K. Dashed lines: the velocities predicted by the best-fit C_{ij} model; Solid squares: experiment data. Error bars smaller than the symbols are not shown.

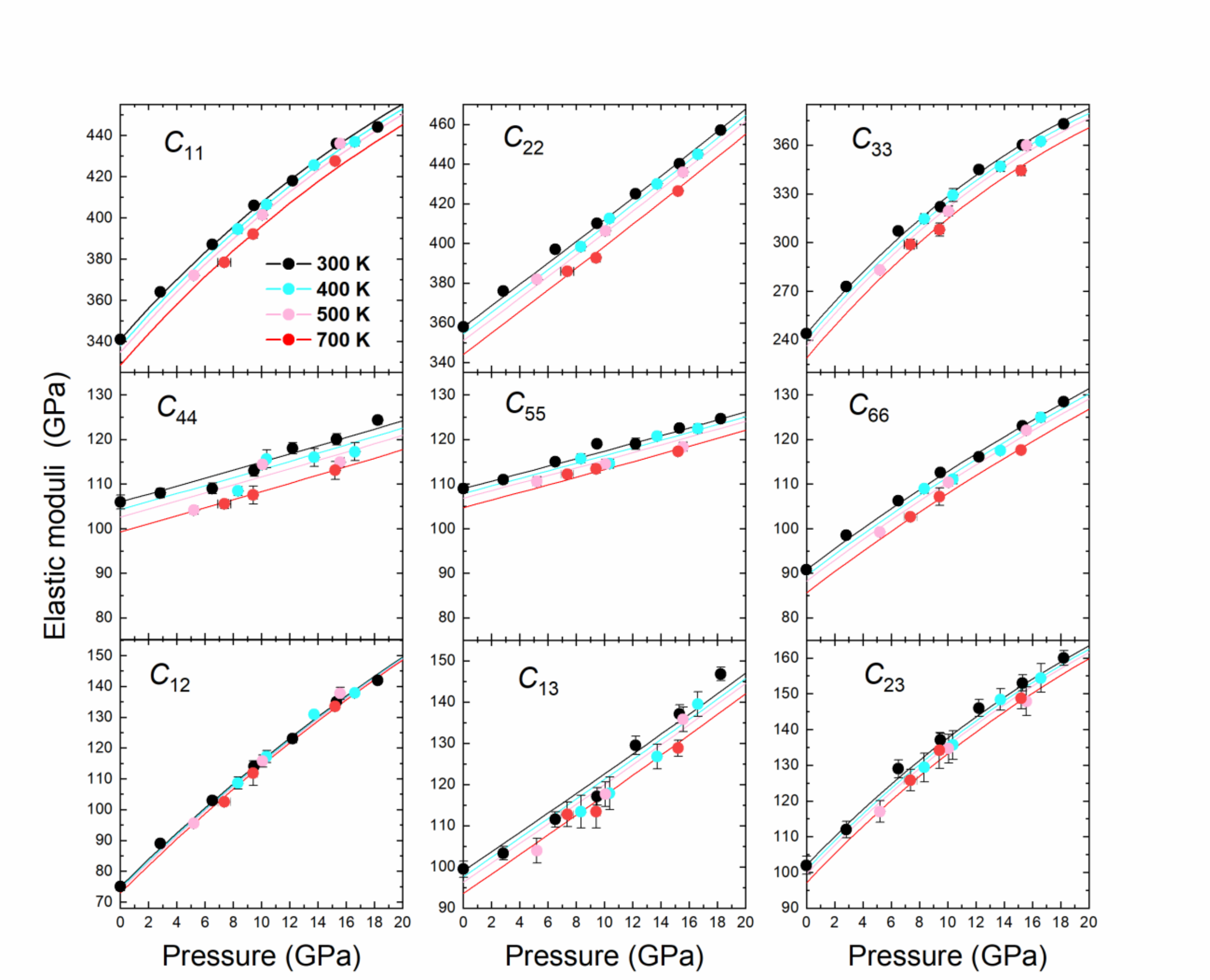

Supplementary Figure 3. Change of the C_{ij}s of the hydrous Fe-bearing wadsleyite sample used in this study as a function of *P* along 300 K, 400 K, 500 K, and 700 K isotherms. Solid lines represent *T*-dependent 3rd or 4th order finite strain EOS fitting results. Data along the 300 K isotherm are adopted from Zhou et al.[1]. Error bars smaller than the symbols are not shown.

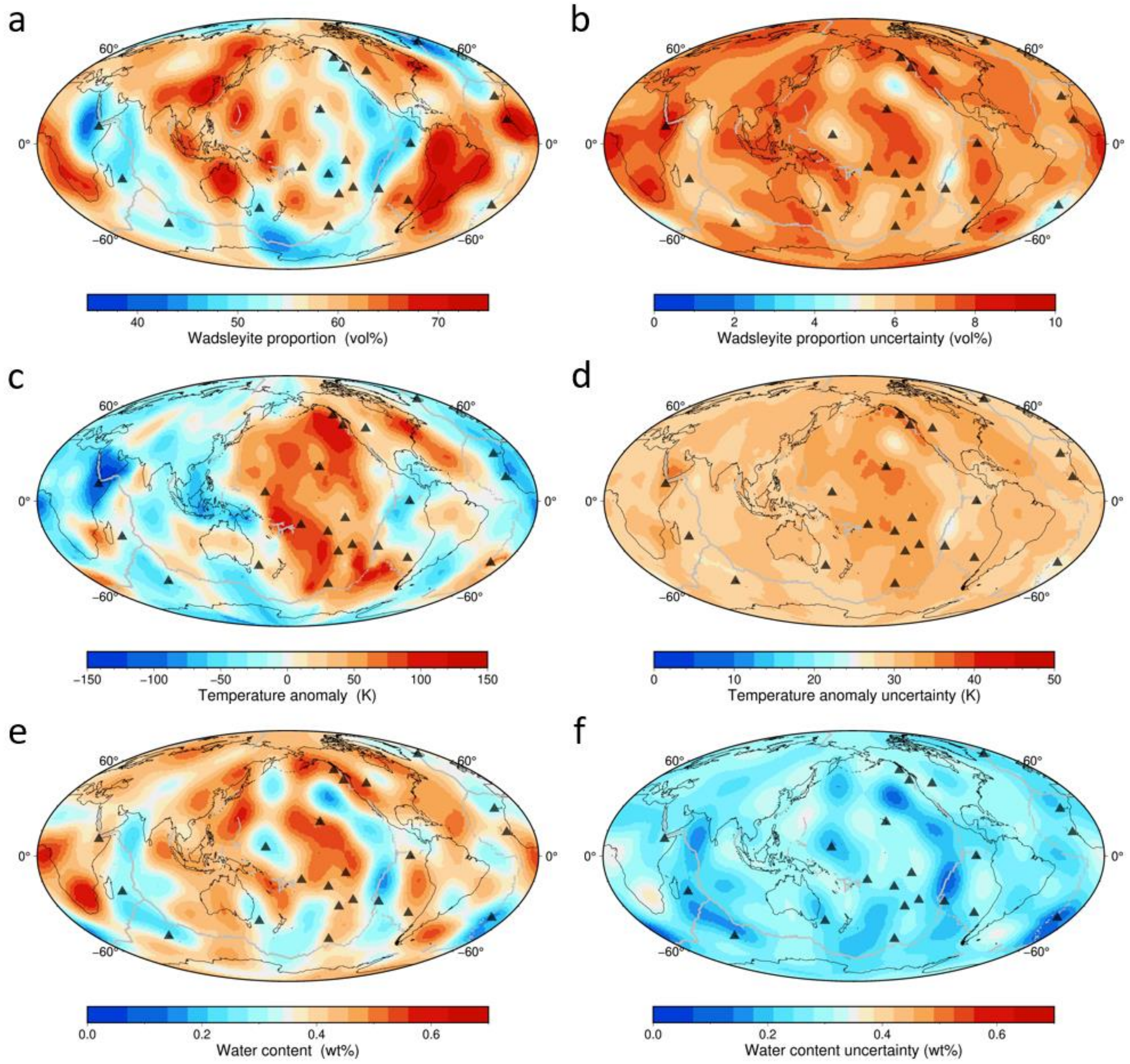

Supplementary Figure 4. Global wadsleyite proportion, temperature anomaly Δ*T*, and water content maps in the upper MTZ with final misfit <1.5, constrained from depth variation of the 410, *V*p and *V*s anomaly at 450 km depth. a global distribution of wadsleyite proportion; **b** global distribution of the uncertainty of wadsleyite proportion; **c** global distribution of Δ*T*; **d** global distribution of the uncertainty of Δ*T*; **e** global distribution of water content; **f** global distribution of the uncertainty of water content. Deeply sourced hotspots[3] are plotted as black triangles.

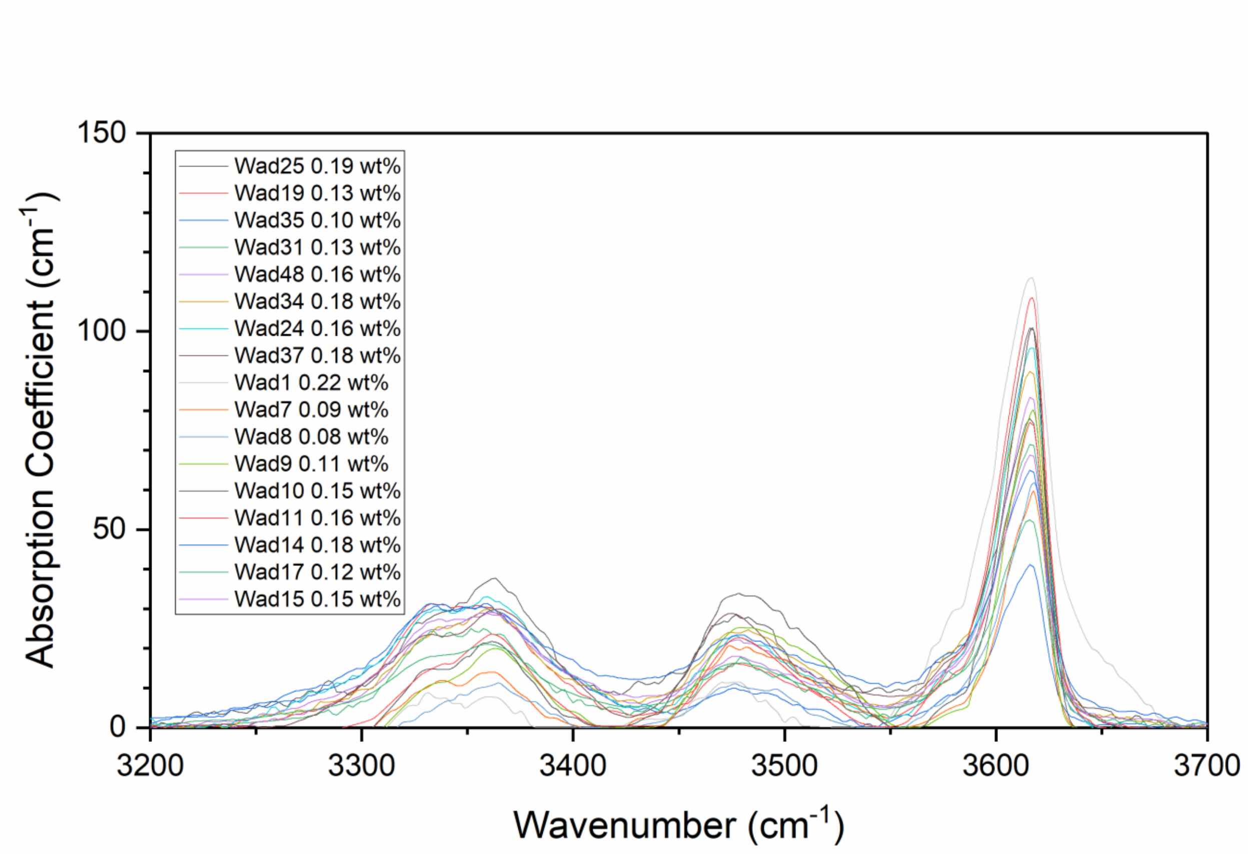

Supplementary Figure 5. Unpolarized FTIR spectra of 17 randomly oriented wadsleyite platelets. The water contents shown in the legend were calculated using the calibration procedure outlined in Libowitzky and Rossman[4].

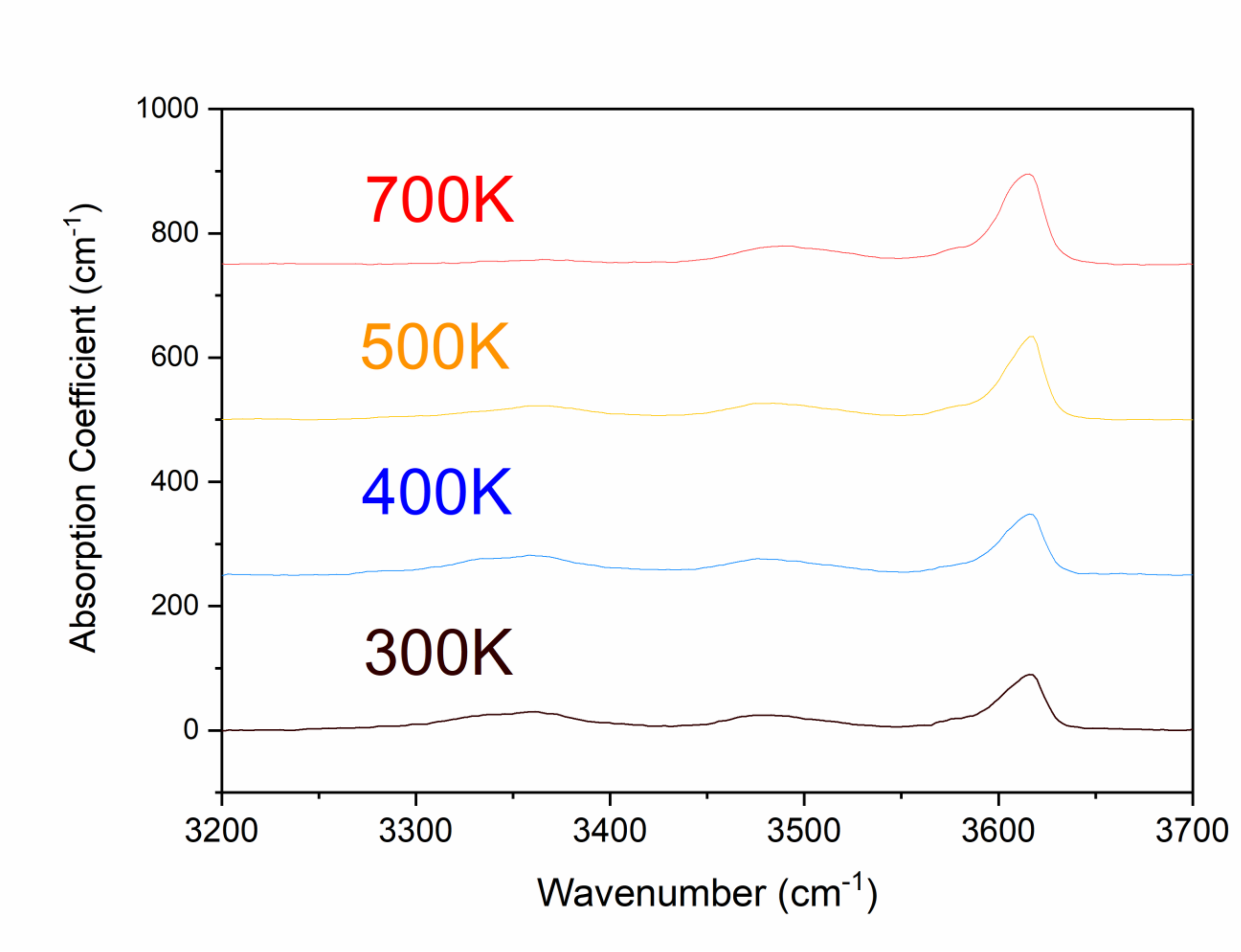

Supplementary Figure 6. FTIR spectra of wadsleyite crystal Wad34 after several heating and cooling cycles. Black line: FTIR spectrum collected before heating; Blue line: FTIR spectrum collected after the sample had been heated at 400 K and then cooled down to 300 K. Yellow line: FTIR spectrum collected after the sample had been heated at 500 K and then cooled down to 300 K. Red line: FTIR spectrum collected after the sample had been heated at 700 K and then cooled down to 300 K.

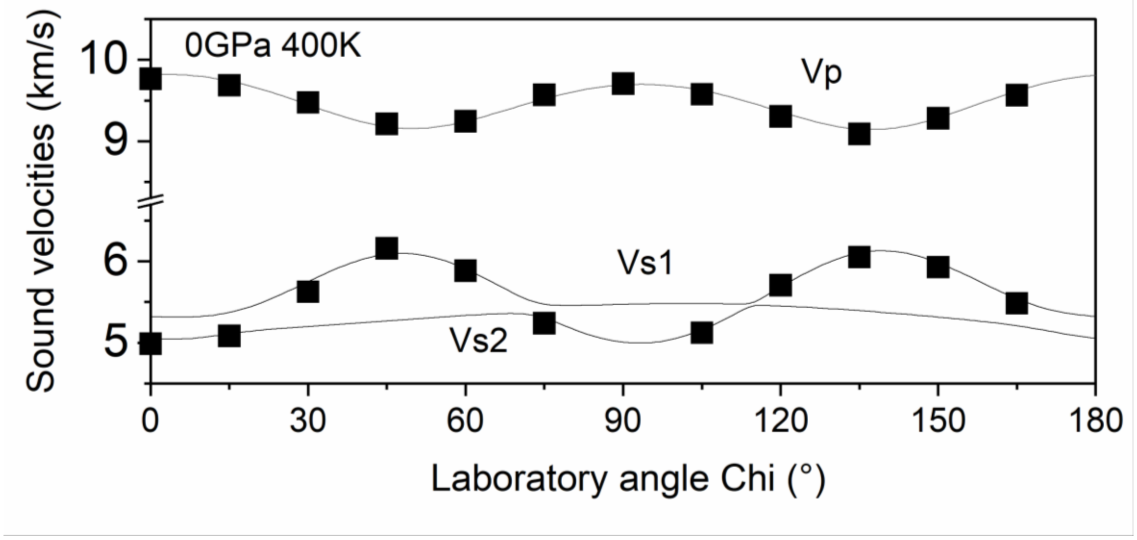

Supplementary Figure 7. Change of velocities for wadsleyite sample Wad9 (0.0748 0.3154 -0.9460) as a function of laboratory angle Chi at 400 K and 0 GPa. Solid lines: the velocities predicted by the best-fit C_{ij} model at 400 K and 0 GPa based on high *P*-*T* experiments in this study; Squares: velocities collected at 400 K and 0 GPa after the completion of the high *P*-*T* experiments. Note, the *V*p and *V*s measurements at 400 K and 0 GPa (squares in this figure) are not utilized to derive the high *P*-*T* C_{ij} model of wadsleyite, which are shown in Supplementary Fig. 3.

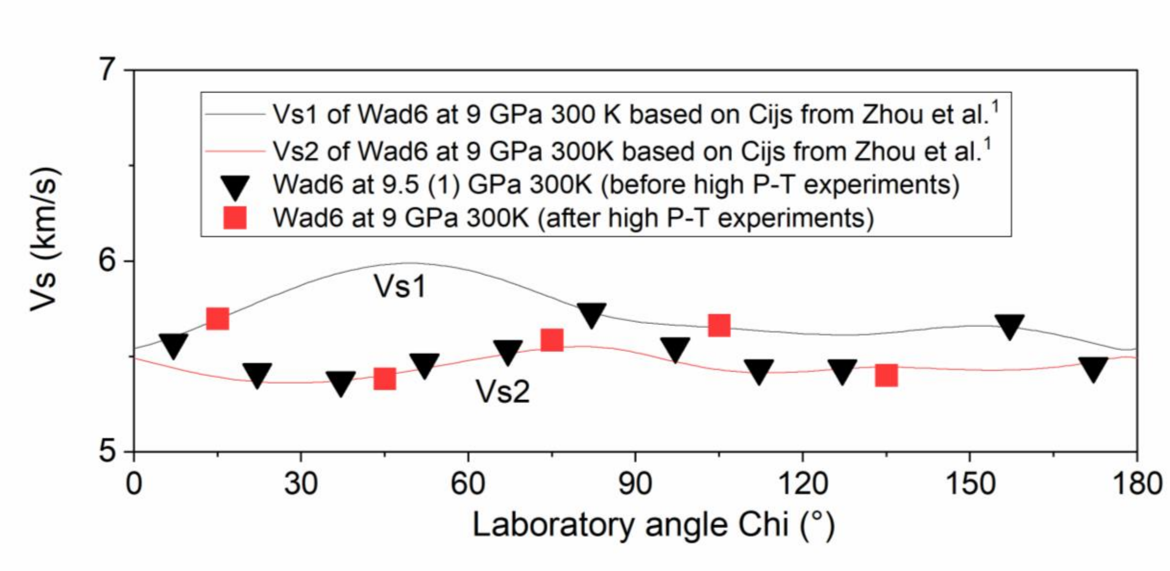

Supplementary Figure 8. Change of *V*s as a function of laboratory angle Chi for wadsleyite sample Wad6 (-0.3420, 0.8718, 0.3506). Solid lines: the velocities predicted by the best-fit C_{ij} model at 300 K and 9 GPa from ambient-*T* high-*P* experiments in Zhou et al.[1] (before high *P*-*T* experiments in this study); Triangles: velocities measured at 300 K and 9.5 (2) GPa in Zhou et al.[1] (before high *P*-*T* experiments in this study); Red squares: velocities measured at 300 K and 9 GPa after the sample has been heated to 700 K and 9.4 GPa in this study.

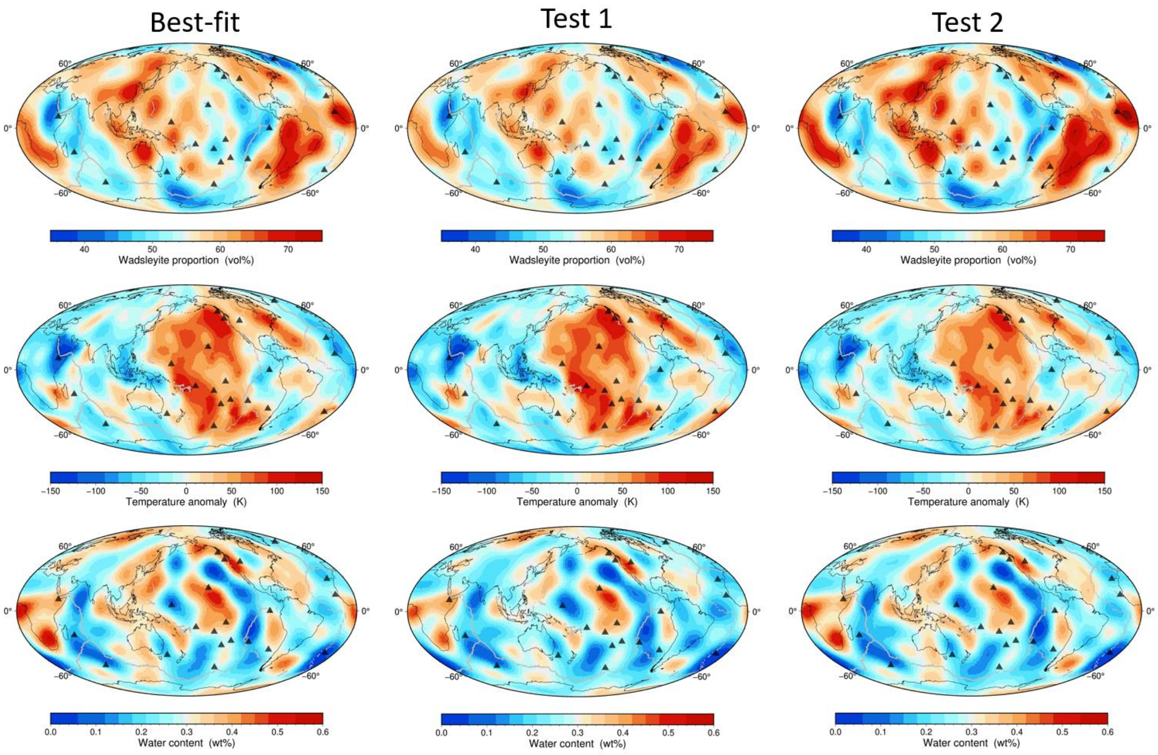

Supplementary Figure 9. Global wadsleyite proportion, ΔT, and water content maps of the upper MTZ based on the 3 different sets of mineral physics parameters shown in Supplementary Table 7.

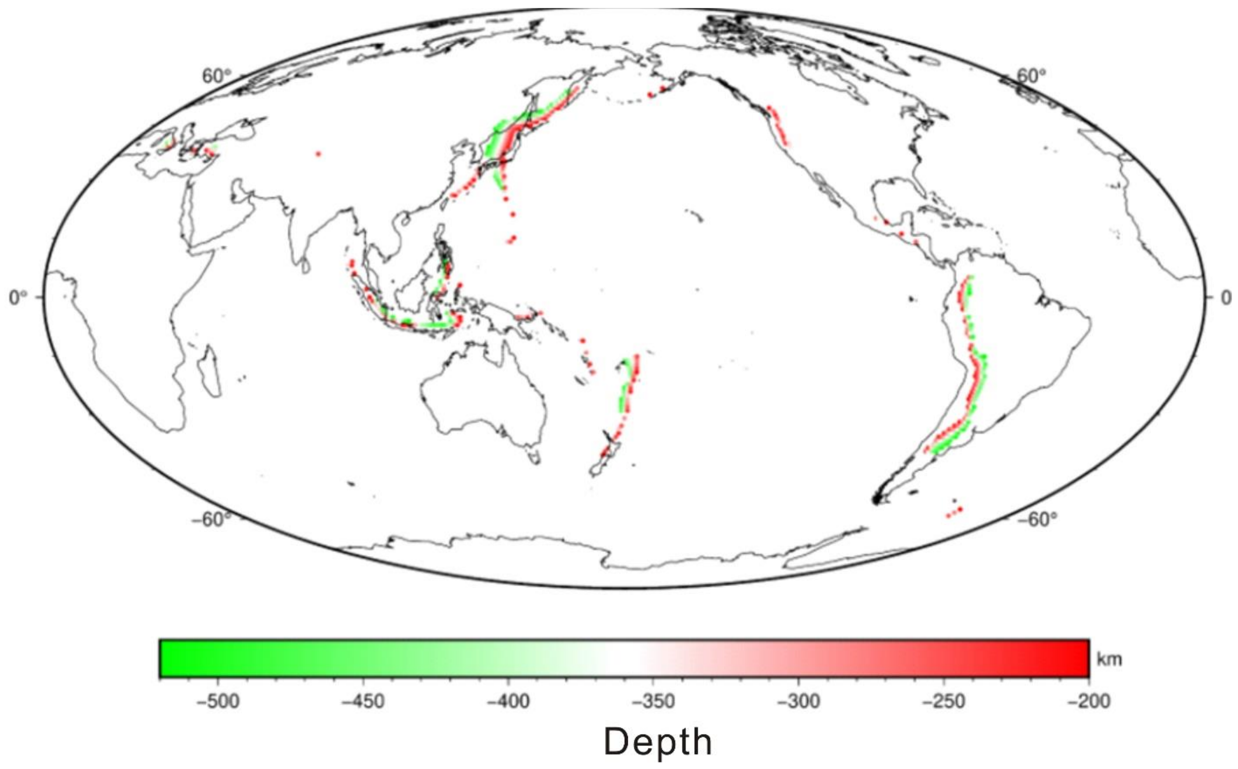

Supplementary Figure 10. Slab locations near the upper MTZ constrained by Slab 2[5]. We used slab locations between 200 and 520 km to ensure the complete coverage of the slabs in the upper MTZ, especially for subduction zones with few earthquakes or sparse sampling in tomography. We also averaged structures within a wide area near each slab (~500 km) since the resolution is relatively low in the upper MTZ.

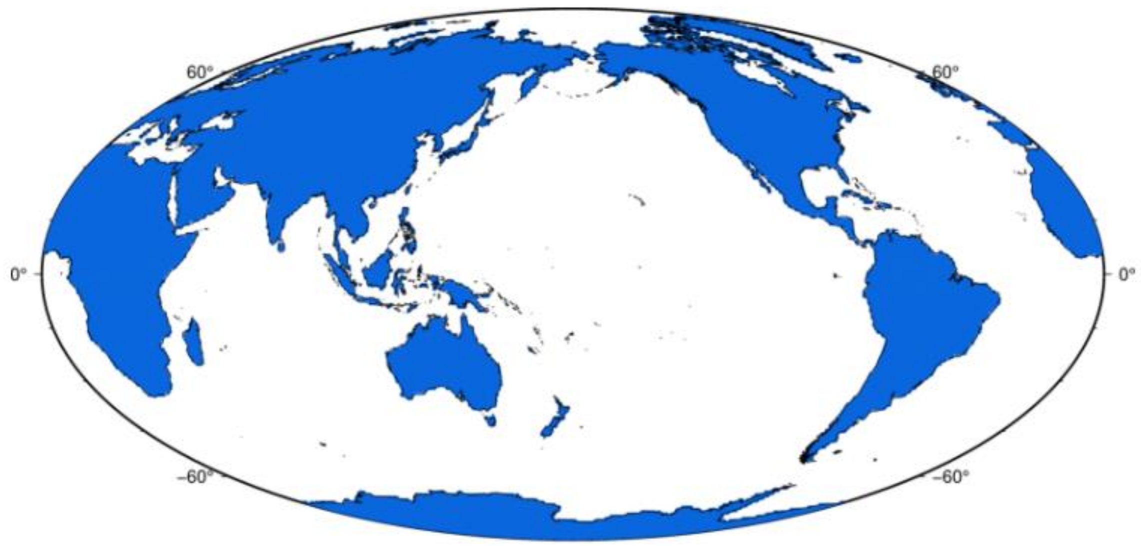

Supplementary Figure 11. Land (blue) and ocean (white) locations downloaded from GMT[6] based on Global Self-consistent, Hierarchical, High-resolution Geography database[7].

Supplementary Table 1. C_{ij}s, *K*s, *G*, *V*p, and *V*s for the hydrous Fe-bearing wadsleyite sample measured in this study.

T	(K)	400	400	400	400	500	500	500	700	700	700
P	(GPa)	8.3(2)	10.4(2)	13.7(2)	16.6(1)	5.2(1)	10.1(3)	15.6(2)	7.4(5)	9.4(3)	15.2(1)
ρ	g cm^{-3}	3.754	3.792	3.851	3.900	3.686	3.780	3.876	3.712	3.750	3.855
C_{11}	(GPa)	394(2)	407(2)	426(1)	437(2)	372(1)	401(2)	436(2)	379(1)	392(2)	428(1)
C_{22}	(GPa)	398(2)	413(2)	430(1)	445(2)	382(1)	406(2)	436(2)	386(1)	393(2)	427(1)
C_{33}	(GPa)	314(3)	329(4)	347(3)	362(2)	283(2)	320(3)	360(3)	299(3)	308(4)	344(3)
C_{44}	(GPa)	109(2)	116(2)	116(1)	117(2)	104(1)	114(1)	115(1)	106(2)	108(2)	113.1(9)
C_{55}	(GPa)	116(1)	115(1)	120.7(9)	122(1)	111(1)	115(1)	118(1)	112.2(8)	113(1)	117.3(7)
C_{66}	(GPa)	109(1)	111(1)	117.5(7)	125(1)	99.3(7)	110.4(9)	122.1(8)	102.7(7)	107(2)	117.6(7)
C_{12}	(GPa)	109(2)	117(2)	131(1)	138(1)	96(1)	116(2)	138(2)	103(1)	112(4)	134(1)
C_{13}	(GPa)	113(4)	118(4)	127(3)	140(3)	104(3)	118(3)	136(3)	113(3)	113(4)	129(2)
C_{23}	(GPa)	129(4)	136(4)	148(3)	154(4)	117(3)	135(4)	148(4)	126(3)	134(5)	149(3)
Ks^{VRH}	(GPa)	201(2)	209(2)	222(2)	233(2)	185(2)	206(2)	229(2)	193(2)	201(2)	224(2)
G^{VRH}	(GPa)	116(1)	119(1)	123(1)	126(1)	110(1)	118(1)	125(1)	111(1)	113(1)	121(1)
*V*p	(km s^{-1})	9.74(2)	9.86(2)	10.02(2)	10.15(1)	9.48(2)	9.80(2)	10.10(2)	9.59(2)	9.69(2)	10.00(1)
*V*s	(km s^{-1})	5.56(2)	5.61(2)	5.66(1)	5.70(1)	5.45(2)	5.58(1)	5.68(1)	5.48(2)	5.50(2)	5.61(1)

Note: VRH refers to Voigt-Reuss-Hill averaging scheme[8].

Supplementary Table 2. Summary of previous high-*T* velocity measurements on different wadsleyite samples, and the experimental result at 300K[1] on the same samples used in this study.

water	Fe#	Ks_0	G_0	$(\partial Ks/\partial P)_{T0}$	$(\partial Ks^2/\partial P^2)_{T0}$	$(\partial G/\partial P)_{T0}$	$(\partial G^2/\partial P^2)_{T0}$	$(\partial Ks/\partial T)_{P0}$	$(\partial G/\partial T)_{P0}$	Experiments	References
wt%		GPa	GPa		GPa^{-1}		GPa^{-1}	GPa K^{-1}	GPa K^{-1}		
0	0	170.7(11)	111.6(5)	4.56(23)	\	1.75(9)	\	−0.013(2)	−0.016(1)	7GPa,873K, UI	[9,10]
0	0	170.2(19)	113.9(7)	\	\	\	\	−0.0171(5)	−0.0157(3)	1bar,660K, RUS	[11]
0	9	165.7(1)	105.66(3)	\	\	\	\	−0.016(3)	−0.012(1)	1bar,318K, RUS	[12]
0	9	165.72(1)	105.43(2)	\	\	\	\	−0.0175(3)	−0.0159(1)	1bar,470K, RUS	[13]
0	13	171.3	108.7	4.74(8)	\	1.52(4)	\	−0.013(1)	−0.0144(8)	12GPa,1073K, UI	[14]
0.73	0	161.5(2)	101.6(1)	4.84(4)	\	1.68(2)	\	-0.013(2)	-0.015(4)	10GPa,600K, UI	[15]
0.15	9.4	165(2)	104(2)	5.2(1)	-0.16(2)	1.9(1)	-0.07(1)	\	\	18GPa, 300K, BS	[1]
0.15	9.4	165(2)	104(2)	5.1(1)	-0.14(2)	1.9(1)	-0.07(1)	−0.018(2)	−0.014(1)	16GPa,700K, BS	This study

UI: Ultrasonic Interferometry

RUS: Resonant Ultrasound Spectroscopy

BS: Brillouin Spectroscopy

Note: The entries at row 7 (reference 1) have been compared with an existing literature focusing on the pressure dependence of the elastic properties of wadsleyite at ambient temperature. The references to those studies can be found in reference 1. The errors shown in this study represent 1 standard deviation.

Supplementary Table 3. C_{ij0}, $(\partial C_{ij}/\partial P)_{T0}$, $(\partial^2 C_{ij}/\partial P^2)_{T0}$, and $(\partial C_{ij}/\partial T)_{P0}$ for the hydrous Fe-bearing wadsleyite samples measured in this study.

	C_{ij0} (GPa)	$(\partial C_{ij}/\partial P)_{T0}$	$(\partial^2 C_{ij}/\partial P^2)_{T0}$ (GPa^{-1})	$(\partial C_{ij}/\partial T)_{P0}$ (GPa K^{-1})
C_{11}	341(1)	7.8(3)	-0.26(4)	-0.029(4)
C_{22}	358(1)	5.37(5)	0	-0.036(3)
C_{33}	244(2)	10.5(2)	-0.46(3)	-0.040(4)
C_{44}	106(2)	0.87(5)	0	-0.018(3)
C_{55}	109(1)	0.84(3)	0	-0.011(2)
C_{66}	90.8(7)	2.43(4)	-0.053(6)	-0.013(1)
C_{12}	75(1)	4.6(2)	-0.11(2)	-0.006(2)
C_{13}	99(2)	2.33(8)	0	-0.019(5)
C_{23}	102(3)	4.2(2)	-0.14(3)	-0.012(3)

Supplementary Table 4. Thermoelastic properties of garnet.

Mineral	Endmember	K_{S0} GPa	G_0 GPa	ρ_0 g cm^{-3}	$(\partial K_S/\partial P)_{T0}$	$(\partial K_S/\partial T)_{P0}$ (GPa K^{-1})	$(\partial G/\partial P)_{T0}$	$(\partial G/\partial T)_{P0}$ (GPa K^{-1})	a_0 (10^{-4} K^{-1})	a_1 (10^{-8} K^{-1})	a_2 (K^{-1})
Garnet	Pyrope[16-20]	171.0(5)	94.9(2)	3.560(2)	4.4(1)	-0.014(3)	1.15(6)	-0.011(2)	0.288	0.2787	-0.5521
	Almandine[16,21]	174.2(12)	94.9(7)	4.319(2)	4.61(14)	-0.0267(7)	1.06(6)	-0.0131(8)	0.26(5)	2.3(14)	0
	Grossular[16,22]	171.2(8)	107.4(2)	3.605(2)	4.47(2)	-0.0138(3)	1.29(5)	-0.0128(2)	0.1951	0.8089	-0.4972
	Mg-majorite[16-20,23]	162.0(5)	86.2(2)	3.560(2)	4.4(1)	-0.014 (3)	1.15(6)	-0.011(2)	0.288	0.2787	-0.5521

Note: In terms of our choice of the source data for calculating the sound velocities of Fe-Ca bearing majoritic garnet in the upper MTZ, our preference was given to the most recent direct sound velocity measurements at simultaneously high P-T conditions. For end member majorite and pyrope, their ambient-condition K_{S0} and G_0 are calculated using the elasticity data of $Maj_{38}Py_{62}$[18], $Maj_{50}Py_{50}$[19,24], $En_{80}Py_{20}$[24]. As suggested by Liu et al.[25], both the velocities and elastic moduli of the garnet within the majorite-pyrope solid solution increase linearly with Al content within analytical uncertainties. Their *P* and *T* derivatives of K_S and *G* are based on the sound velocity data collected on a pyrolitic garnet ($Maj_{50}Py_{18}Gro_{25}Alm_6$) by Irifune et al.[26] up to ~18 GPa and 1673 K, which are close to the *P-T* conditions we explored in this study. The thermoelastic properties of the almandine end member are adopted from the most recent high *P-T* sound velocity measurements up to 19 GPa and 1700 K by Arimoto et al.[21]. For the grossular end member, the values presented in this table are from the most recent high *P-T* elasticity measurements up to 10 GPa and 1000 K by Gwanmesia et al.[15]

Supplementary Table 5. Water contents of the 17 different wadsleyite crystals determined using unpolarized FTIR with two different calibration methods. The water contents of sample Wad1-Wad17 have been determined and reported in Zhou et al.[1]. We reported the values calculated based on the Libowitzky and Rossman[4] calibration in the main text to be consistent with previous studies[27,28].

Sample Name	Water content (wt %)	
	Libowitzky and Rossman[4] calibration	Deon et al.[29] calibration
Wad25	0.19	0.17
Wad19	0.13	0.13
Wad35	0.10	0.11
Wad31	0.13	0.11
Wad48	0.16	0.14
Wad 34	0.18	0.15
Wad24	0.16	0.15
Wad37	0.18	0.16
Wad1[1]	0.22	0.12
Wad7[1]	0.09	0.08
Wad8[1]	0.08	0.06
Wad9[1]	0.11	0.10
Wad10[1]	0.15	0.12
Wad11[1]	0.16	0.12
Wad14[1]	0.18	0.17
Wad15[1]	0.15	0.14
Wad17[1]	0.12	0.11
Average	0.15 (4)	0.13 (3)

Supplementary Table 6. Sound velocities of pyrolite at 15.2 GPa and 1870 K[30] calculated using 2 different methods. The thermoelastic parameters used to determine these values are reported in Supplementary Tables 2, 4.

Pyrolite	Velocities by volume-weighted elastic moduli (km s^{-1})	Direct volume-weighted velocities (km s^{-1})	Difference
*V*p	9.521	9.527	6 m s^{-1}, 0.1%
*V*s	5.147	5.153	6 m s^{-1}, 0.1%

Supplementary Table 7. Two different sets of mineral physics parameters used in the modeling tests described in Supplementary Discussion 1, and their differences from the best-fit parameters.

Parameters	a	b	c	d	A	B	C	D	e	f
**Best-fit**	-0.0027	-0.0028	0.0397	0.9750	-0.0040	-0.0045	0.0868	0.4850	0.107	20
**Test 1**	-0.0026	-0.0029	0.0358	0.9439	-0.0036	-0.0054	0.0957	0.4266	0.102	19
**difference**	-4%	5%	-10%	-3%	-11%	18%	10%	-12%	-4%	-5%
**Test 2**	-0.0028	-0.0027	0.0341	1.0444	-0.0036	-0.0049	0.0741	0.5405	0.122	25
**difference**	3%	-3%	-14%	7%	-11%	9%	-15%	11%	14%	25%

Supplementary References

1 Zhou, W.-Y. *et al.* The Water-Fe-Pressure dependent single-crystal elastic properties of wadsleyite: Implications for the seismic anisotropy in the upper Mantle Transition Zone. *Earth and Planetary Science Letters* **565**, 116955 (2021).

2 Katsura, T., Yoneda, A., Yamazaki, D., Yoshino, T. & Ito, E. Adiabatic temperature profile in the mantle. *Physics of the Earth and Planetary Interiors* **183**, 212-218, doi:10.1016/j.pepi.2010.07.001 (2010).

3 Courtillot, V., Davaille, A., Besse, J. & Stock, J. Three distinct types of hotspots in the Earth's mantle. *Earth and Planetary Science Letters* **205**, 295-308 (2003).

4 Libowitzky, E. & Rossman, G. R. An IR absorption calibration for water in minerals. *American Mineralogist* **82**, 1111-1115 (1997).

5 Hayes, G. P. *et al.* Slab2, a comprehensive subduction zone geometry model. *Science* **362**, 58-61 (2018).

6 Wessel, P. *et al.* The generic mapping tools version 6. *Geochemistry, Geophysics, Geosystems* **20**, 5556-5564 (2019).

7 Wessel, P. & Smith, W. H. A global, self‐consistent, hierarchical, high‐resolution shoreline database. *Journal of Geophysical Research: Solid Earth* **101**, 8741-8743 (1996).

8 Hill, R. Elastic properties of reinforced solids: some theoretical principles. *Journal of the Mechanics and Physics of Solids* **11**, 357-372 (1963).

9 Li, B., Liebermann, R. C. & Weidner, D. J. P‐V‐Vp‐Vs‐T measurements on wadsleyite to 7 GPa and 873 K: Implications for the 410‐km seismic discontinuity. *Journal of Geophysical Research: Solid Earth* **106**, 30579-30591 (2001).

10 Liu, W., Kung, J. & Li, B. Elasticity of San Carlos olivine to 8 GPa and 1073 K. *Geophysical Research Letters* **32** (2005).

11 Isaak, D. G. *et al.* The elastic properties of β-Mg2SiO4 from 295 to 660K and implications on the composition of Earth's upper mantle. *Physics of the Earth and Planetary Interiors* **162**, 22-31, doi:10.1016/j.pepi.2007.02.010 (2007).

12 Katsura, T. *et al.* Temperature derivatives of elastic moduli of (Mg0. 91Fe0. 09) 2SiO4 modified spinel. *Physics of the Earth and Planetary Interiors* **124**, 163-166 (2001).

13 Mayama, N. *et al.* Temperature dependence of elastic moduli of β‐(Mg, Fe) 2SiO4. *Geophysical research letters* **31** (2004).

14 Liu, W., Kung, J., Li, B., Nishiyama, N. & Wang, Y. Elasticity of (Mg0.87Fe0.13)2SiO4 wadsleyite to 12GPa and 1073K. *Physics of the Earth and Planetary Interiors* **174**, 98-104, doi:10.1016/j.pepi.2008.10.020 (2009).

15 Gwanmesia, G. D. *et al.* The Elastic Properties of β-Mg2SiO4 Containing 0.73 wt.% of H2O to 10 GPa and 600 K by Ultrasonic Interferometry with Synchrotron X-Radiation. *Minerals* **10**, 209 (2020).

16 Fei, Y. Thermal expansion. *Mineral physics and crystallography: a handbook of physical constants* **2**, 29-44 (1995).

17 Irifune, T. *et al.* Sound velocities of majorite garnet and the composition of the mantle transition region. *Nature* **451**, 814-817, doi:10.1038/nature06551 (2008).

18 Liu, J., Chen, G., Gwanmesia, G. D. & Liebermann, R. C. Elastic wave velocities of pyrope–majorite garnets (Py62Mj38 and Py50Mj50) to 9 GPa. *Physics of the Earth and Planetary Interiors* **120**, 153-163 (2000).

19 Sinogeikin, S. V. Elasticity of Majorite and a Majorite-Pyrope solid solution to high pressure: Implications for the Transition Zone. *Geophysical Research Letters* **29**, doi:10.1029/2001gl013937 (2002).

20 SUZUKI, I. & ANDERSON, O. L. ELASTICITY AND THERMAL EXPANSION OF A NATURAL GARNET UP TO 1, 000K. *Journal of Physics of the Earth* **31**, 125-138 (1983).

21 Arimoto, T., Gréaux, S., Irifune, T., Zhou, C. & Higo, Y. Sound velocities of Fe3Al2Si3O12 almandine up to 19 GPa and 1700 K. *Physics of the Earth and Planetary Interiors* **246**, 1-8, doi:10.1016/j.pepi.2015.06.004 (2015).

22 Gwanmesia, G. D., Wang, L., Heady, A. & Liebermann, R. C. Elasticity and sound velocities of polycrystalline grossular garnet (Ca3Al2Si3O12) at simultaneous high pressures and high temperatures. *Physics of the Earth and Planetary Interiors* **228**, 80-87 (2014).

23 Hao, M. *et al.* The seismically fastest chemical heterogeneity in the Earth's deep upper mantle—implications from the single-crystal thermoelastic properties of jadeite. *Earth and Planetary Science Letters* **543**, 116345 (2020).

24 Sinogeikin, S. V. & Bass, J. D. Elasticity of pyrope and majorite–pyrope solid solutions to high temperatures. *Earth and Planetary Science Letters* **203**, 549-555 (2002).

25 Liu, Z. *et al.* Influence of aluminum on the elasticity of majorite-pyrope garnets. *American Mineralogist: Journal of Earth and Planetary Materials* **104**, 929-935 (2019).

26 Irifune, T. *et al.* Sound velocities of majorite garnet and the composition of the mantle transition region. *Nature* **451**, 814-817 (2008).

27 Mao, Z. *et al.* Single-crystal elasticity of wadsleyites, β-Mg2SiO4, containing 0.37–1.66 wt.% H2O. *Earth and Planetary Science Letters* **268**, 540-549, doi:10.1016/j.epsl.2008.01.023 (2008).

28 Buchen, J. *et al.* High-pressure single-crystal elasticity of wadsleyite and the seismic signature of water in the shallow transition zone. *Earth and Planetary Science Letters* **498**, 77-87, doi:10.1016/j.epsl.2018.06.027 (2018).

29 Deon, F., Koch-Müller, M., Rhede, D. & Wirth, R. Water and Iron effect on the P-T-x coordinates of the 410-km discontinuity in the Earth upper mantle. *Contributions to Mineralogy and Petrology* **161**, 653-666, doi:10.1007/s00410-010-0555-6 (2010).

30 Ishii, T., Kojitani, H. & Akaogi, M. Phase relations and mineral chemistry in pyrolitic mantle at 1600–2200 °C under pressures up to the uppermost lower mantle: Phase transitions around the 660-km discontinuity and dynamics of upwelling hot plumes. *Physics of the Earth and Planetary Interiors* **274**, 127-137, doi:10.1016/j.pepi.2017.10.005 (2018).
