# Supplementary material for: Constraining composition and temperature variations in the mantle transition zone
Source: Nat Commun. 2022 Mar 1;13:1094. doi: 10.1038/s41467-022-28709-7 (PMC8888558; doi:10.1038/s41467-022-28709-7)
Supplement: Supplementary file 3 — Description of Additional Supplementary Files [file 41467_2022_28709_MOESM3_ESM.pdf]

Description of Additional Supplementary Files

File name: Supplementary Data 1
Description: This file presents global models of wadsleyite proportion, temperature anomaly, and water content in the upper Mantle Transition Zone with misfit less than 1.

File name: Supplementary Data 2
Description: This file presents global models of wadsleyite proportion, temperature anomaly, and water content in the upper Mantle Transition Zone with misfit less than 1.5.
